# Synergy of bacteriophage depolymerase with host immunity rescues sepsis mice infected with hypervirulent *Klebsiella pneumoniae* of capsule type K2

**DOI:** 10.1080/21505594.2024.2415945

**Published:** 2024-10-21

**Authors:** Deyi Zhao, Miran Tang, Zhexiao Ma, Panjie Hu, Qingxia Fu, Zhuocheng Yao, Cui Zhou, Tieli Zhou, Jianming Cao

**Affiliations:** a School of Laboratory Medicine and Life Science, Wenzhou Medical University, Wenzhou, China; b Department of Clinical Laboratory, The First Affiliated Hospital of Wenzhou Medical University, Wenzhou, China; c Key Laboratory of Clinical Laboratory Diagnosis and Translational Research of Zhejiang Province, Wenzhou, Zhejiang, China

**Keywords:** Hypervirulent *K. pneumoniae*, phage depolymerase, capsular polysaccharide, host immunity, anti-virulence agent, sepsis

## Abstract

The hypervirulent *Klebsiella pneumoniae* (hvKp) with K1 and K2 capsular types causes liver abscess, pneumonia, sepsis, and invasive infections with high lethality. The presence of capsular polysaccharide (CPS) resists phagocytic engulfment and contributes to excessive inflammatory responses. Bacteriophage depolymerases can specifically target bacterial CPS, neutralizing its defense. Based on our previous research, we expressed and purified a bacteriophage depolymerase (Dep1979) targeting hvKp with capsule type K2. Interestingly, although Dep1979 lacked direct bactericidal activity *in vitro*, it exhibited potent antibacterial activity *in vivo*. Low-dose Dep1979 (0.1 mg/kg) improved the 7-day survival of immunocompetent mice to 100%. Even at 0.01 mg/kg, mice achieved 100% survival at 5 days, although efficacy sharply declined at doses as low as 0.001 mg/kg. Following Dep1979 treatment, reduced expression of inflammatory factors and no apparent tissue damage were observed. However, therapeutic efficacy significantly diminished in immunosuppressed mice. These findings underscore the critical role of Dep1979 in disarming CPS, which synergizes with host immunity to enhance antibacterial activity against hvKp.

## Graphical abstract

**Figure:**
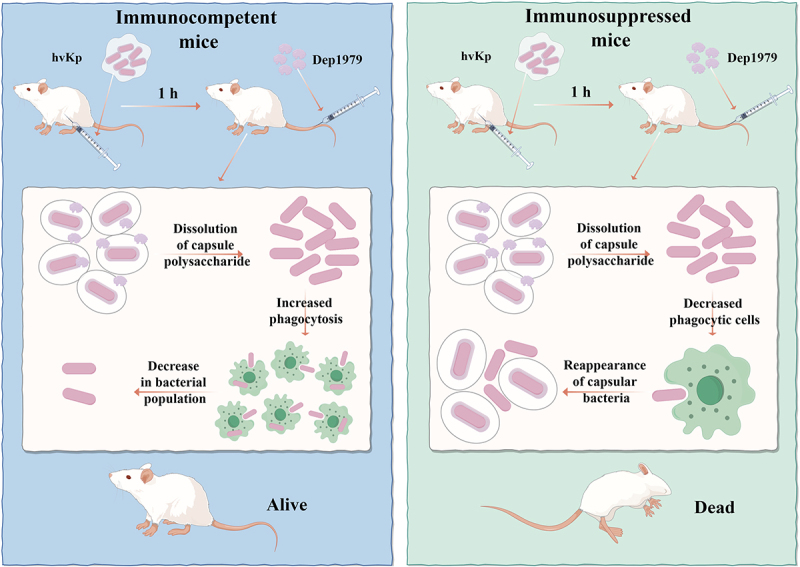
After treatment with Dep1979, the hypervirulent *K. pneumoniae* FK1979 lost its CPS, making it more susceptible to clearance by immune cells such as macrophages and neutrophils, which led to a reduction in bacterial numbers and increased survival of the mice. In contrast, immunocompromised mice had reduced numbers and functions of phagocytes and overall immune capacity, which hindered the effective clearance of CPS-deficient FK1979, resulting in bacterial proliferation exceeding the host’s clearance rate. Additionally, as Dep1979 was metabolized and consumed by the bacteria, its effective concentration in the body decreased. Consequently, FK1979 with CPS reappeared, leading to increased virulence and ultimately causing the death of the mice.

## Introduction

*K. pneumoniae*, a common gram-negative facultative anaerobe, has emerged as a causative agent of nosocomial infections. The clonal spread of virulence and antibiotic resistance plasmids has led to the increasing emergence of hvkp, carbapenem-resistant *K. pneumoniae*, and carbapenem-resistant hvKp worldwide [1–3]. Hvkp-induced infections, such as pneumonia, sepsis, meningitis, and pyogenic liver abscess, are associated with high mortality rates, and traditional antibiotics often struggle to promptly address the septic shock triggered by these bacteria [4,5]. According to the latest WHO report, “Antimicrobial Resistance, Hypervirulent *Klebsiella pneumoniae* – Global Situation,” the global risk of infection and transmission associated with hvKp has been assessed as moderate.

The presence of *K. pneumoniae* CPS, a crucial virulence factor, has been linked to approximately 130 different *K. pneumoniae* capsule serotypes (K-types) [6]. Of these, K1, K2, K4, K5, K20, and K54 are closely associated with high virulence, with the majority confined to K1 or K2 [7,8]. CPS allows resistance to serum killing, enhances invasiveness, and protects bacteria from phagocytosis by host immune cells, enabling them to survive even after ingestion. Therefore, despite *in vitro* susceptibility tests indicating sensitivity, the high virulence and ability of hvKp to evade host immune defenses often lead to adverse patient outcomes [9–12]. Among them, the interactions between hvKp and the host, especially the shock induced by the cytokine storm mediated by hvKp-triggered immune responses, maybe a key factor in its adverse outcomes [13,14].

Bacteriophages possess bactericidal capabilities against specific bacteria, offering advantages such as avoiding indiscriminate harm to the normal human microbiota, being unaffected by antibiotic resistance, and degrading biofilms [15]. However, these advantages come with limitations, including a narrow host range, the need for personalized treatment plans (which complicates empirical treatment), and the rapid development of bacterial tolerance [16]. Nevertheless, successful treatment in specific clinical cases has renewed interest in phage therapy [17,18]. In addition, certain phages encode depolymerases that disrupt the capsule of *K. pneumoniae*, facilitating bacteriophage entry into cells by depolymerizing extracellular polysaccharides. While the direct bactericidal activity of bacteriophages is often emphasized, the importance of capsule depolymerization in treating hvKp infections is sometimes overlooked [19]. Disarming the hvKp capsule increases its susceptibility to host immune recognition, phagocytosis by host immune cells, and complement-mediated killing [20,21]. Thus, depolymerases may offer a new perspective for hvKp treatment.

Our previous study isolated a bacteriophage (ΦFK1979) targeting the K2-type hvKp FK1979 from hospital wastewater. Based on its phenotype and genomic characteristics, we inferred its potential to produce a capsule-specific depolymerase [16]. In the current study, we expressed and purified depolymerase1979 (Dep1979). We established infection models using hvkp FK1979 in immunocompetent and immunosuppressed mice and treated them with Dep1979 or traditional antimicrobial drugs. Immunocompetent mice responded effectively to Dep1979 treatment at doses as low as 0.1 mg/kg. However, Dep1979 failed to rescue immunosuppressed mice infected with the same bacterial load, suggesting that the *in vivo* efficacy of phage depolymerase is closely related to host immune status. More comprehensive treatment plans may be required for patients with severely compromised immune function.

## Methods

### Bacterial strains

The *K. pneumoniae* strains used in this study were isolated from clinical nonrepetitive strains at the Wenzhou Medical University First Affiliated Hospital (FK1979, FK4176, FK5537, and FK6048). The strain with the number xxxx is denoted as FKxxxx. Based on positive string tests (>5 mm) on blood agar plates, all four K-type strains were identified as K2 type.

### Production and purification of depolymerase

The tested hvKp FK1979 and its specific phage ΦFK1979 were isolated and characterized in our previous work [16]. As previously described [22], the ORF1 of phage ΦFK1979 encoding K2 CPS-specific depolymerase was amplified and sequenced. The full-length ORF1 gene segment and truncated signal peptide gene segment were ligated into the vector PET28a-XAA and transformed into DH5α for identification. Positive clone plasmids were extracted after plating the transformed and amplified DH5α on LB agar plates containing 50 μg/mL kanamycin and incubating at 37°C for 12–16 h. These plasmids were then introduced into protein-expressing bacterial cells. When the cell density reached an OD_600_ of 0.6, the cells were induced with 0.4 M IPTG overnight at 20°C. Subsequently, the cells were centrifuged at 2000 × g for 8 minutes, and the cell culture was collected. The cell culture was thoroughly resuspended in buffer A, with 0.1 M PMSF added at a 1:500 (v/v) ratio per milliliter of culture. After adding lysozyme E to a final concentration of 1 μg/mL, the mixture was sonicated in an ice-water bath for 1 h. The lysate was centrifuged for 30 minutes at 15,000 × g. His-XAA tagged protein purification was performed by applying the supernatant to an open column filled with nickel-chelating agarose resin, which had been pre-equilibrated with buffer A. The target protein was eluted using different concentrations of buffer B. The concentration of the purified His-XAA-Dep1979 recombinant protein was then quantified, and TEV protease (at a ratio of 1:50 of the total protein) was added to the protein for overnight cleavage at 4°C. The cleaved target protein was repurified using a nickel affinity column, eluted with different concentrations of buffer B, desalted, and finally quantified. The SDS-PAGE analysis of purified Dep1979 is shown in (Fig. S1).

### Depolymerase host profile and capsule digestion activity

To determine the specificity of the depolymerase for the K2 capsule and evaluate its range of activity, spot tests were performed. LB agar plates were covered with 5 mL of LB 50% agar medium containing 1 × 10^6^ CFU/mL of FK1979, FK4176, FK5537, and FK6848, respectively, and incubated at 37°C for 2 h. Subsequently, 5 µL of depolymerase (at concentrations of 5, 0.5, 0.05, 0.005, 0.0005, and 0.00005 μg) was spotted on the plates. The plates were then incubated at 37°C for 24 h, and the size of the halo formed after capsule digestion was observed.

### Growth curve

The growth curve experiment aimed to determine whether bacterial growth was affected by depolymerase treatment. LB broth (20 mL) with varying doses of depolymerase at a density of 1 × 10^6^ CFU/mL was used for the initial inoculation of FK1979. Following inoculation, the cultures were incubated at 37°C and 180 rpm in a temperature-controlled incubator. At 0, 2, 4, 6, 12, and 24 h, 200 μL of the bacterial suspension was taken out of the shaking flask and transferred into a 96-well plate. The microplate reader was used to measure the absorbance at 600 nm. Each sample was measured in triplicates and averages of absorbance values were used for analysis.

### Time-dependent killing assay

The time-dependent killing assay, with some modifications [23], involved adding depolymerase at concentrations of 100, 10, and 1 μg/mL to LB broth containing 1 × 10^6^ CFU/mL of FK1979. The mixture was co-cultured at 37°C with agitation at 200 rpm for 24 h, with PBS as the control group. At each time point (0, 2, 4, 6, 8, 12, and 24 h), bacterial suspensions were serially diluted 10-fold with PBS, and 10 µL of each dilution was plated on LB agar plates. After incubation at 37°C for 16–18 h, colony counting was performed where individual colonies were uniformly distributed, with a detection limit of 2 log10 CFU/mL. Each sample was measured in triplicates and averages of absorbance values were used for analysis.

### Extraction and quantification of capsule

Uronic acid was extracted and quantified as described previously [3,24]. In brief, PBS was used as a reference, and 500 μL of bacterial cultures were cultivated for 6 h before being treated with various Dep1979 concentrations (1 μg/mL, 10 μg/mL, and 100 μg/mL) for 2 h. Afterward, 100 μL of 1% ZWITTERGENT 3–14 detergent in 100 mm citric acid was added to the mixture, which was incubated for 20 minutes at 50°C. Following bacterial precipitation, 300 μL of the supernatant was combined with 1.2 mL of 100% ethanol, incubated for 20 minutes at 4°C, and centrifuged for 5 minutes at 14,000 × g. The solid particles were dehydrated and then mixed with 200 μL of distilled water. To this mixture, 1.2 mL of a solution containing 12.5 mm of sodium tetraborate sulfate was added. The resulting mixture was heated to 100°C for 5 minutes and immediately cooled on ice for 5 minutes. Next, a 30-μL aliquot of a solution containing 0.15% 3-phenylphenol in 0.5% NaOH was introduced. The absorbance at 520 nm was measured after incubating the solution at room temperature for 5 minutes. The glucuronic acid content was determined from a standard curve of glucuronic acid and expressed as μg/10^8^ CFU. Results were presented as mean and standard deviation of data of three independent experiments.

### Biofilm formation inhibition assays

Biofilm formation inhibition assays were conducted as previously described [25]. Briefly, four K2 *K. pneumoniae* strains were tested as test strains. The experimental group was treated with different concentrations of Dep1979: 1 μg/mL, 10 μg/mL, and 100 μg/mL. PBS served as the control group. The cultures were placed in an environment with a controlled temperature of 37°C for 24 h. For the assay, 100 µL of the drug solution was mixed with 100 µL of the bacterial suspension in 96-well microplates. After incubation, the excess medium was removed, and planktonic bacteria were rinsed with PBS to facilitate dehydration for water fixation. Next, 170 µL of 1% crystal violet (CV) dye (Beijing Solarbio Biotechnology Co., LTD., China) was added to each well. The plates were incubated at 37°C for 15 minutes and washed twice with PBS. The bound CV was then dissolved with 170 µL of absolute ethanol, and absorbance was measured at 595 nm using a microplate reader (Multiskan FC). Each sample was measured in triplicates and averages of absorbance values were used for analysis.

### Phagocytosis assay

The phagocytosis assay, using the mouse macrophage line RAW264.7, was designed to evaluate the phagocytic ability of macrophages after depolymerase treatment against FK1979 [26]. RAW264.7 murine macrophages (Procell Life Science and Technology Co., Ltd., China) were cultured in DMEM medium supplemented with 10% heat-inactivated fetal bovine serum (GIBCO, Life Technologies) and 100 U/mL penicillin +100 μg/mL streptomycin (Gibco™, Life Technologies, Waltham, MA) at 37°C in a 5% CO_2_ incubator. The FK1979 strain was cultured with shaking at 37°C for 5 h until it reached the logarithmic growth phase. The bacteria were then exposed to Dep1979 at a concentration of 10 μg/mL for 2 h. Following this, the bacteria were resuspended in a DMEM culture medium, with PBS serving as the reference group. A single-cell layer of 1 × 10^6^ CFU/mL macrophages was seeded in a six-well plate. A 10:1 ratio of FK1979 bacteria to cells was achieved by adding the treated FK1979, followed by incubation at 37°C for 2 h. Following three washes, DMEM culture media supplemented with kanamycin (100 μg/mL) was introduced and incubated at 37°C for 1 h. Following three subsequent washes, 1 mL of a 0.5% solution of Triton X-100 was introduced, and the cells were repeatedly pipetted to ensure thorough cell lysis. The phagocytic rate was calculated by determining the number of bacteria recovered. Results were presented as mean and standard deviation of data of three independent experiments.

### Scanning electron microscopy (SEM) and transmission electron microscopy (TEM) of the bacterial capsule

The FK1979 strain was inoculated into 20 mL of LB broth and incubated at 37°C with continuous shaking at 180 rpm for 5 h until it reached the logarithmic growth phase. The bacterial suspension was subsequently diluted to a concentration of 1 × 10^7^ CFU/mL. The experimental group was treated with Dep1979 at a concentration of 10 μg/mL, while PBS served as the control. The cultures were incubated at 37°C while shaking at 180 rpm for 3 h. After incubation, the cultures were washed once with PBS and centrifuged at 1500 × g for 5 minutes. The supernatant was discarded, and the bacterial pellets were resuspended in 200 μL of PBS. The bacterial suspension was carefully added dropwise onto glass silicon chips, allowing the liquid to settle and dry completely. The fixed bacteria were then treated with a 2.5% glutaraldehyde solution overnight. Following fixation, the samples were dried using a critical point drying apparatus. The dried samples were mounted onto sample holders using conductive carbon adhesive, and platinum was sputtered onto them for approximately 120 seconds using an ion-sputtering instrument. SEM was to observe and analyze the bacterial morphology.

In the TEM experiment, the conditions and duration for depolymerase treatment of FK1979 were the same as those in the SEM experiment. After centrifuging and collecting the FK1979 cells treated with Dep1979 and PBS, we resuspended the cell pellet in 1.5-mL EP tubes containing 2.5% glutaraldehyde. It was incubated at 4°C for 12 h. The glutaraldehyde fixative was discarded and the sample was gently rinsed three times with PBS buffer. The sample was fixed with 1% osmium tetroxide solution for 1 h. The osmium tetroxide waste was removed and the sample was rinsed three times with 0.1 M PBS buffer (pH 7.4). The sample was gradually dehydrated using a series of ethanol solutions: 30%, 50%, 70%, and 90%, with each step lasting 15 minutes. It was dehydrated twice with 100% ethanol for 20 minute each time. Finally, it was dehydrated twice with 100% acetone for 20 minutes each time. The sample was infiltrated with a 1:1 mixture of acetone and embedding resin at 37°C for 3 h. It was then infiltrated with a 1:3 mixture of acetone and embedding resin at 37°C for 4 h. Next, the sample was incubated with pure embedding resin at 37°C overnight. The pure resin was poured into embedding molds and the sample was placed into the molds. The sample was cured in a 70°C oven. The embedded sample was polymerized in a 70°C oven for 12 h. After polymerization, the resin block was removed for further processing. Using an ultramicrotome, the resin block was sectioned into ultrathin sections of 70–90 nm. The sections were collected on copper grids. The sections were stained with uranyl acetate for 15 minutes, followed by lead citrate staining for 10 minutes. After drying, the bacterial morphology was observed under TEM and images were captured.

### Construction of mouse infection model and treatment with depolymerase

Male ICR mice aged 6–8 weeks (Vital River, Zhejiang, China) were used as experimental mice. Animal research was conducted following ethical standards and approved by the Ethics Committee of the First Affiliated Hospital of Wenzhou Medical University (Approval Number: WYYY-AEC-2022-047), following the “Wenzhou City Experimental Animal Welfare and Ethical Standards.” Mice were housed in groups of five per cage, identified with cage cards and tail marks using a permanent marker, and maintained under a 12-h light-dark cycle with ad libitum access to food and water. Mice were anesthetized with 2.0%–5.0% isoflurane gas for approximately 5 minutes, achieving adequate anesthesia as indicated by loss of consciousness and lack of response to noxious stimuli (e.g. toe pinch). At the end of the experiment, mice were humanely euthanized by carbon dioxide asphyxiation, followed by cervical dislocation.

Based on previous studies, significant differences in susceptibility to respiratory and systemic symptoms in sepsis models have been observed between male and female mice, with male mice being more suitable for constructing a sepsis model [27]. A mouse model of systemic sepsis was established by intraperitoneal injection of FK1979 bacterial suspension grown to the logarithmic phase, with treatment administered via tail vein injection. We conducted survival curve experiments and mouse organ bacterial load counting experiments in both immunocompetent and immunosuppressed mice. Mice were randomly divided into four groups (*n* = 10): Control group (PBS), Model group (1 × 10^7^, 1 × 10^6^, 1 × 10^5^, 1 × 10^4^, and 1 × 10^3^ CFU FK1979), Treatment group (0.01, 0.1, and 1 mg/kg Dp1979), and Antibiotic group (64 mg/kg IMP). Immunocompromised mice were induced by continuous injection of 150 mg/kg/day cyclophosphamide for 4 days. Macrophage-depleted mice were established by injecting 200-μL clodronate liposomes into the tail vein for 3 consecutive days, followed by an additional 100 μL clodronate liposomes on the fourth day [28]. The experimental grouping was the same as that for immunocompetent mice [29]. For organ bacterial load counting experiments (*n* = 5), mice were anesthetized at 24 hours postinfection (hpi). Blood was collected from the orbit, followed by euthanasia. Organs such as the heart, liver, spleen, lung, and kidney were collected after disinfection. If mice died within 24 h, these procedures were conducted preemptively when the mice were approaching death. After weighing, 1 mL of PBS was added to grind and homogenize the mouse tissues. The dilution method for blood and tissue homogenates was consistent with that used in the previous time-dependent killing assay. Additionally, 10 μL of undiluted blood and tissue homogenates were used for spot plate counting. Due to the use of alcohol to disinfect the mouse skin during dissection, potential contamination of the collected tissues and organs cannot be ruled out. Therefore, for counts of less than 5 CFU in the undiluted blood and tissue homogenates, the values were recorded as zero. In all mouse experiments, a single drug treatment was administered 1 h after bacterial infection, and the number of mouse deaths was recorded every 12 h for survival curve correlation experiments.

### Determination of histopathology and inflammatory cytokines

For the histopathological tissue section experiment, the experimental mouse groups were the same as those used for organ bacterial load counting in immunocompetent mice, with samples collected at 24 hpi. For mice that died prematurely, samples were collected as soon as possible. Collected organs (liver, spleen, lungs, and kidneys) were fixed in 4% paraformaldehyde solution, embedded in paraffin, and sectioned at 5 μm. The tissue sections were stained with hematoxylin and eosin for histological examination, and pathological sites were observed and photographed under a microscope. For inflammatory factor detection, blood samples were collected from the eye orbit at 8 hpi and centrifuged at 1500 × g for 15 minutes to collect the serum. The concentrations of inflammatory factors (TNF-α, IL-6, and IL-1β) were measured using ELISA assay kits.

### Statistical analysis

The data are presented as the mean ± standard deviation and were analyzed using one-way and two-way analysis of variance (ANOVA) with GraphPad Prism 10.2.0 (GraphPad Software, La Jolla, California, USA). Differences were considered statistically significant at *p < 0.05*.

## Results

### The growth of bacteria in vitro was not inhibited by Dep1979

**Figure 1.**
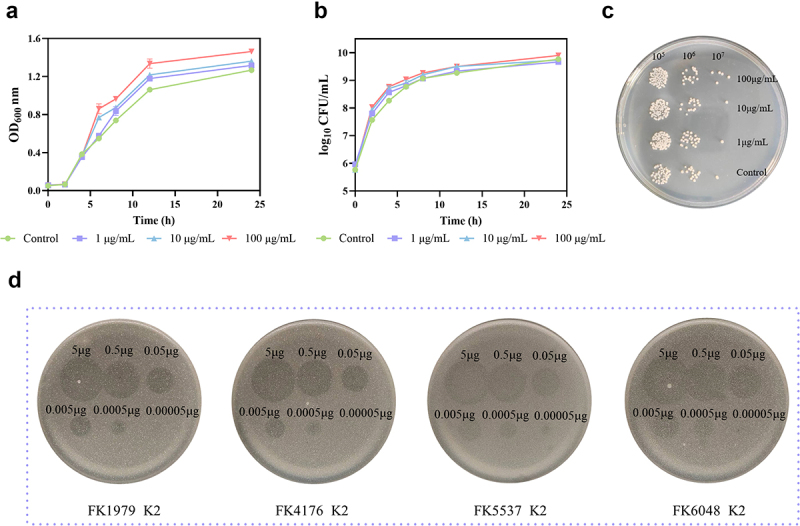
*In vitro* antimicrobial activity and host profile of Dep1979. (a) The 24-h growth curves of FK1979 were treated with Dep1979 or PBS. (b) The 24-h time killing kinetics of FK1979 treated with Dep1979 or PBS. (c) Bacterial loads were enumerated after FK1979 treatment with Dep1979 or PBS for 2 h. (d) Dep1979 displayed capsule lysis specificity and different concentration digestion activity against four *K. pneumoniae* strains (K-2 type).

Because of the host-targeting property of phage depolymerase, we assessed the host spectrum range of Dep1979. Results indicated that Dep1979 specifically targets *K. pneumoniae* of the K2 capsule type (Figure 1d). However, it remains unclear whether capsule destruction affects bacterial proliferation. The *in vitro* antibacterial activity of Dep1979 was evaluated using time-dependent killing and growth curve assays. Growth curve experiments revealed that compared with the control group, the growth of FK1979 was not inhibited within 24 h of Dep1979 treatment; instead, there was a slight increase in growth rate (Figure 1a). Similarly, time-dependent killing assays showed no significant impact on FK1979 growth over 24 h with Dep1979 treatment. At a concentration of 100 μg/mL, bacterial growth rate slightly increased compared to the control group, although this difference was not statistically significant (Figure 1b). After 2 h of treatment, the 100 μg/mL group also exhibited a marginally higher bacterial count than the control (Figure 1c). Our results suggest that Dep1979 lacks antibacterial activity and does not inhibit bacterial growth *in vitro*. However, whether Dep1979 can accelerate the *in vitro* proliferation of bacteria after depolymerase treatment needs to be further investigated.

### Dep1979 digests CPS, inhibits biofilm formation, and increases macrophage phagocytosis

**Figure 2.**
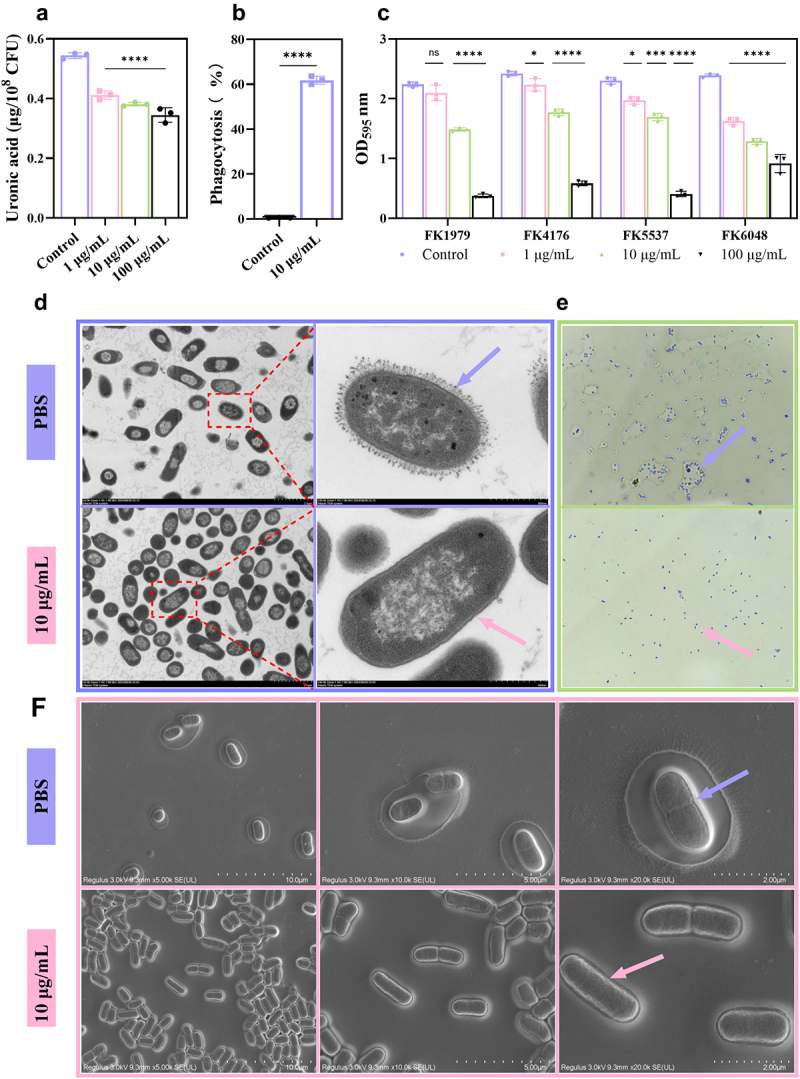
(a) Quantification of uronic acid after Dep1979 treatment. (b) Biofilm inhibition assay with Dep1979 against four *K. pneumoniae* of capsule type K2 isolates. (c) The phagocytosis rate of FK1979 by macrophages treated with PBS or Dep1979. (d) TEM images of bacterial capsules treated without or with Dep1979. (e) After Dep1979 treatment, exopolysaccharide digestion was performed by rapid crystal violet staining and FK1979. (f) SEM images of bacterial capsules treated without or with Dep1979. ns, not statistically significant; *p < 0.05; ***p < 0.001; ****p < 0.0001 were analyzed by one-way ANOVA and two-way ANOVA multiple comparisons and t-tests.

Increasing evidence shows that most hvKp strains exhibit high CPS yield and a high-viscosity phenotype. The presence of CPS is closely linked to bacterial resistance to phagocytosis, complement-mediated killing, and inflammatory responses. Therefore, exploring Dep1979’s ability to digest CPS is crucial; this was assessed through exopolysaccharide (uronic acid) quantification in our study. Dep1979 exhibited significant hydrolytic activity against FK1979 capsular polysaccharides at different concentrations compared to the control group (Figure 2a). After CV staining, the clear mucus surrounding the Dep1979-treated bacteria vanished, presenting as single, uniformly distributed cells, as indicated by a pink arrow under the microscope. Conversely, the PBS control group exhibited a substantial amount of clear mucus enveloping the bacteria, forming clumps (purple arrow in Figure 2e). To observe changes in CPS, we collected SEM and TEM images under Dep1979 treatment and PBS treatment conditions. Figure 2f shows that bacteria in the PBS control group were enveloped in mucus, with obvious capsule coverage on the bacterial surface and a thick capsule layer around the cell body, as indicated by a purple arrow. In contrast, Dep1979-treated bacteria displayed roughened surfaces, clear gaps between bacteria, and the disappearance of the white capsule layer surrounding the bacteria, as indicated by a pink arrow. To further observe the changes in FK1979 capsular polysaccharides after Dep1979 treatment, we collected TEM images (Figure 2d). At 30,000× magnification, compared to the PBS-treated group (indicated by the purple arrow), the disappearance of the CPS layer around the bacterial cells was evident in the Dep1979-treated group (indicated by the pink arrow) compared to the PBS-treated group (indicated by the purple arrow). The ability of Dep1979 to degrade the capsule was visually confirmed by the optical microscope images.

Additionally, given that CPS presence is linked to biofilm formation, its disruption by Dep1979 May potentially inhibit bacterial biofilm formation. Results indicated that Dep1979 reduced the biofilm formation ability of all tested strains (Figure 2c) in a concentration-dependent manner. To further explore whether bacteria were more easily engulfed by phagocytes after capsule removal, mouse macrophage RAW264.7 was employed as the experimental cell. Results showed the phagocytosis efficiency of RAW264.7 macrophages treated with Dep1979 increased from 0.5% to 62%, with the number of phagocytosed bacteria increasing by two orders of magnitude (Figure 2b). Our findings demonstrate that CPS significantly increases hvKp resistance to phagocytes and that its disruption can significantly enhance bacterial phagocytosis efficiency by macrophages.

### Dep1979 rescued immunocompetent septic mice but failed to rescue immunosuppressed infected mice

**Figure 3.**
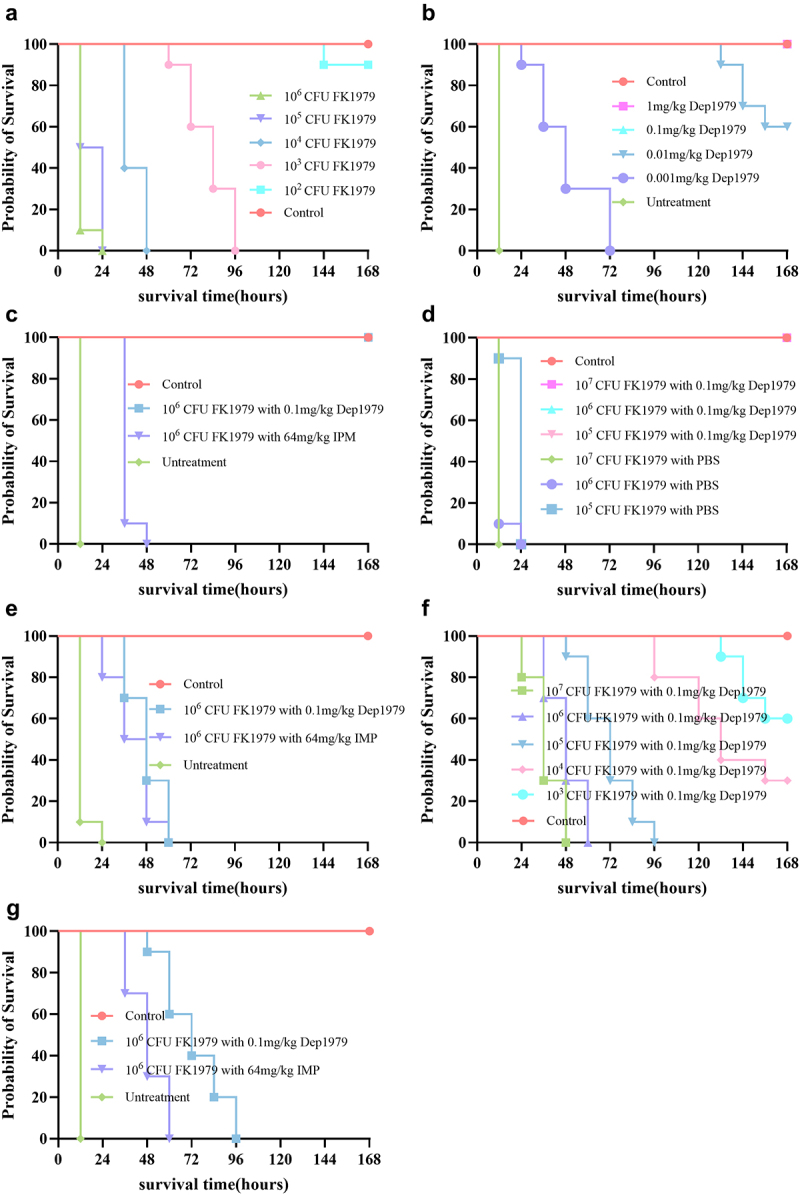
(a) Minimum lethal dose (MLD) of FK1979 within 7 days. (b) The lowest effective therapeutic dose of Dep1979 against 106 CFU FK1979 infection. (c) The survival rate of immunocompetent mice treated with Dep1979, IMP and untreated, and blank groups. (d) Survival of mice with different initial amounts of infected bacteria treated with the same dose of Dep1979. (e) The survival rate of immunosuppressed mice treated with Dep1979, IMP and untreated, and blank groups. (f) Survival of immunosuppressed mice infected with different amounts of bacteria and treated with the same dose of Dep1979. (g) The survival rates of macrophage-depleted mice in the Dep1979, IMP, untreated, and control groups.

**Figure 4.**
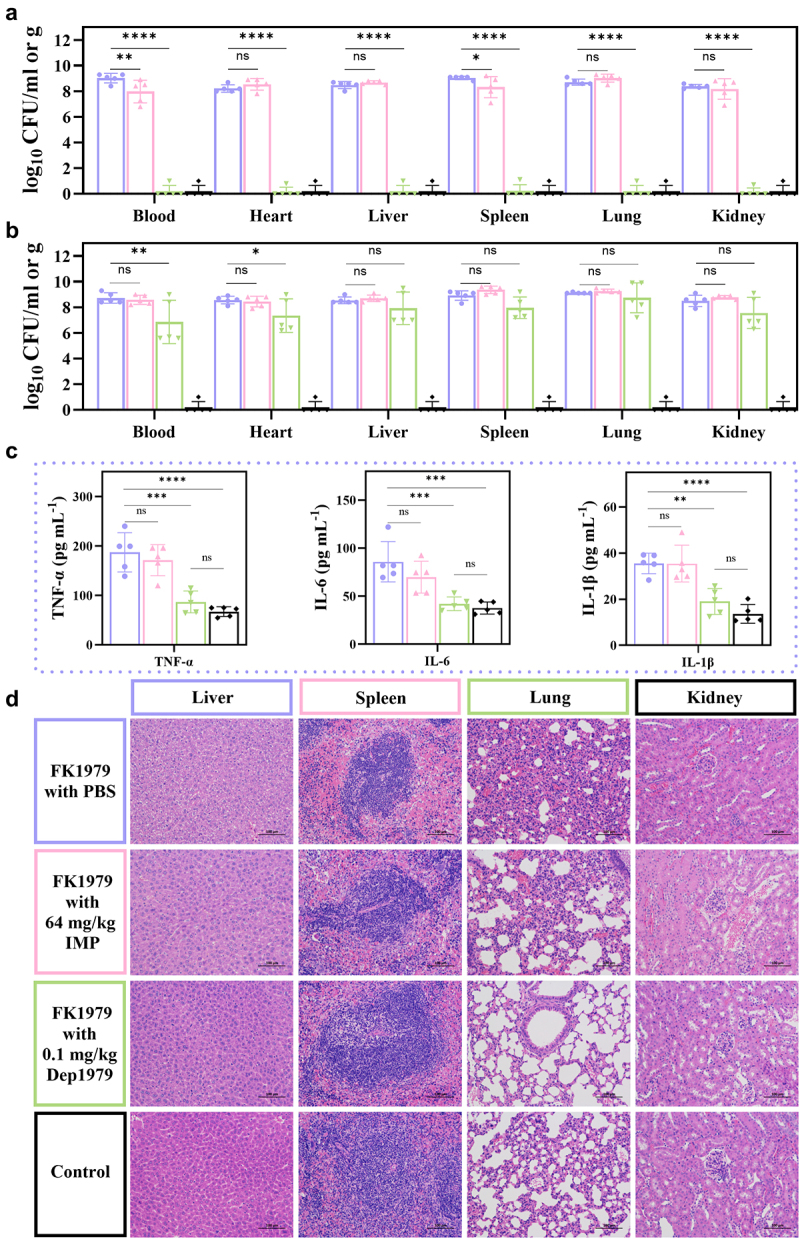
The treatment conditions in a, b, and c are the same as in d, with corresponding colors. (a) The results of bacterial load count in the blood and organs of immunocompetent mice in different treatment groups. (b) The results of bacterial load count in the blood and organs of immunosuppressed mice in different treatment groups. (c) Plasma inflammatory factor concentrations in different treatment groups of immunocompetent mice. (d) Pathological sections of the organs from immunocompetent mice. ns, not statistically significant; *p < 0.05; **p < 0.01; ***p < 0.001, and ****p < 0.0001 were analyzed by one-way ANOVA and two-way ANOVA multiple comparisons.

As a protease-like drug, bacteriophage depolymerase Dep1979’s stability in serum and *in vivo* distribution are crucial for its internal application. We established a sepsis mouse model via acute intraperitoneal infection to assess Dep1979’s in vivo efficacy. The minimum lethal dose causing 100% mortality in mice within 7 days was 10^3^ CFU (Figure 3a). Untreated mice infected with 10^6^ CFU of FK1979 exhibited elevated body temperature, rapid breathing, and increased heart rate within 12 h, followed by acute death. Furthermore, autopsies revealed splenomegaly, lung congestion, and abundant bacterial presence in the blood and various organs. However, Dep1979 treatment increased the survival rate to 100% in immunocompetent mice over 7 days. Compared to the commonly used antibiotic imipenem (64 mg/kg), a single high dose of imipenem prolonged the survival time of infected mice (36 and 48 h) and reduced bacterial loads in some mice’s blood and organs. However, it did not alter the overall survival outcome (Figure 3c and 4a). Compared to using bacteriophage depolymerase, multiple intermittent treatments with imipenem may be necessary to improve the survival rate of mice. Unexpectedly, Dep1979 at a therapeutic dose as low as 0.1 mg/kg achieved 100% survival in immunocompetent mice over 7 days. At a dose of 0.01 mg/kg, it increased the survival rate to 100% within 5 days, demonstrating high antibacterial activity and *in vivo* bioavailability. However, the efficacy of Dep1979 declined sharply when the dose was reduced to 0.001 mg/kg. Meanwhile, we investigated the efficacy of Dep1979 in immunocompetent mice infected with varying initial doses of FK1979 at the same treatment dosage (0.1 mg/kg). The results indicated that even with an initial infection dose of 10^7^ CFU, the 7-day survival rate of mice remained 100% (Figure 3b,d).

Our *in vitro* antibacterial activity studies suggest that Dep1979 lacks antibacterial activity. Macrophages treated with Dep1979 showed increased phagocytosis of FK1979. Our research and previous studies suggest that Dep1979 May exert *in vivo* antibacterial activity by reducing bacterial resistance to innate immunity and enhancing the phagocytic activity of macrophages and neutrophils against the bacteria [30,31]. To validate this hypothesis, we tested Dep1979 in immunosuppressed mice created by cyclophosphamide injection. The results indicated that although Dep1979 treatment prolonged the survival time of infected mice compared to immunocompetent mice, it could not rescue immunosuppressed infected mice, who still had high bacterial counts in the blood and various organs (Figure 4b). It may be necessary to consider the compromised immune system of immunosuppressed mice, which reduces their resistance to FK1979 and increases mortality. Notably, even immunocompetent mice experience 100% mortality within 48 hpi with 10^5^ and 10^4^ CFU of FK1979. Interestingly, when the initial infection dose was reduced to 10^5^ and 10^4^ CFU, Dep1979 treatment significantly increased the 7-day survival rate of infected immunosuppressed mice. This improvement might be due to residual and newly produced immune cells in immunosuppressed mice exerting their effects *in vivo*. The imipenem (64 mg/kg) treatment did not improve the 7-day survival rate or reduce bacterial counts in the blood and organs of infected immunosuppressed mice (Figure 3a,e,f). Cyclophosphamide’s nonspecific cytotoxicity toward immune cells reduces neutrophil and macrophage counts, both of which are crucial for phagocytosing invading hvKp [32]. Given that Dep1979 treatment enhances macrophage-mediated phagocytosis of FK1979 *in vitro*, we used clodronate liposomes to deplete macrophages and establish a macrophage-depleted mouse infection model. This allowed us to investigate the effect of macrophage-specific depletion on Dep1979 efficacy. Interestingly, after Dep1979 treatment, the 7-day survival rate of macrophage-depleted mice reached 100% (Figure 3g), indicating that even under conditions of macrophage depletion and immunosuppression, CPS-deficient FK1979 can still be effectively cleared. These results suggest that anti-virulence therapy remains essential for treating immunocompromised patients infected with hvKp. Furthermore, single bactericidal drug treatment may be ineffective for treating hvKp infections. Traditional antibiotics combined with anti-virulence agents and immunomodulators may be more beneficial in treating hvKp infections.

### Dep1979 alleviated inflammatory response and tissue/organ damage in mice

The concentrations of inflammatory factors (TNF-α, IL-6, and IL-1β) in the serum and histopathological sections of the mice were assessed to determine the impact of phage depolymerase treatment on reducing inflammation and tissue damage. Results revealed significantly lower levels of these inflammatory factors in the Dep1979 treatment group than in the untreated group, with no notable difference observed compared to the PBS group. Conversely, there were no significant differences in inflammatory factor concentrations between the imipenem- and untreated groups, aligning with the survival outcomes and bacterial load in the imipenem-treated group (Figure 4c).

To intuitively assess Dep1979’s efficacy, we analyzed histopathological changes in mouse tissues (liver, spleen, lung, and kidney). Within 24 hpi, immunocompetent mice in the untreated and imipenem-treated groups exhibited varying degrees of organ and tissue damage, including renal interstitial edema and spleen congestion, with the most notable changes observed in alveolar wall thickening and lung bronchi. Conversely, the Dep1979-treated group showed no significant histopathological damage (Figure 4d). These results, consistent with the levels of inflammatory factors and bacterial load in mouse organs, further demonstrate Dep1979’s effectiveness in treating infections caused by K2 capsular type hvKp infections.

## Discussion

The hypermucoviscous phenotype in *K. pneumoniae* is closely associated with the presence of the RmpA-encoding plasmid [33]. Subsequent studies have shown that strains with a string test result ≥5 mm and carrying *IUC*, *iro*, *prmpA*, and *prmpA2* are more likely to be hvKp. However, some strains with hypermucinous phenotypes were hypervirulent. Similarly, some hypervirulent strains showed negative results in the string test [34]. Definitive assessment is often based on survival rates in mouse infection models and clinical outcomes in patients [35–37]. Xueting et al. showed that *K. pneumoniae* strains with K1, K2, and K20 serotypes exhibited near 100% mortality within 48 h in mouse infection models, with significantly higher blood bacterial loads compared to other capsular types of Kp [38]. In our study, the experimental strain FK1979, a K2 hvKp, demonstrated a remarkably low 100% lethal dose of 10^3^ CFU in the mouse infection model within 7 days. At an infective dose of 10^6^ CFU, mice were rapidly killed within 12 hours.

HvKp exhibits various virulence factors, including capsules, exotoxins, and adhesion factors, which aid in evading host immune system attacks, rapidly invading host tissues, and causing severe infections [39]. In response, the hosts initiate a series of immune responses to clear pathogens. However, immune responses triggered by hvKp infections may lead to severe cytokine storms – an extreme immune response phenomenon often accompanied by excessive inflammation that can result in tissue and organ damage [40,41]. Overactivated inflammatory responses can cause vascular leakage, cellular damage, and organ dysfunction, potentially leading to serious respiratory issues. Furthermore, cytokine storms may extend beyond the immediate site of infection, potentially triggering systemic inflammatory reactions and leading to multiple organ dysfunction syndrome, a critical complication that threatens patient survival [42–44]. Given the complexity of cytokine storms, treatment is challenging. Traditional antibiotics may reduce bacterial loads but may not fully control cytokine storms [9,45]. Hence, a comprehensive approach that combines antibiotics with immunomodulators may be necessary. Our findings indicate that Dep1979 effectively reduces inflammatory marker expression and ameliorates the detrimental effects on essential organs resulting from severe sepsis induced by hvKp.

The presence of CPS enhances the formation of *K. pneumoniae* biofilms, which protect the bacteria in harsh environments, leading to increased antimicrobial use and exacerbated antimicrobial resistance [9,46]. Removing CPS can significantly inhibit biofilm formation and improve the efficacy of antibiotics, but the reduction of exopolysaccharides may cause transient resistance to peptide antibiotics such as colistin [46–49]. CPS also stimulates macrophages to produce TNF-α and IL-6 through TLR4/ROS/PKC-δ/NF-κB, TLR4/PI3-kinase/AKT/NF-κB, and TLR4/MAPK signaling pathways [50]. Targeting the capsule may provide a new strategy for treating hvKp (or classic *K. pneumoniae* with a capsule) infection [12,38,51]. However, compared to the broad-spectrum antimicrobial activity of traditional antibiotics, specific depolymerases offer potential advantages in precision medicine. They can accurately target and kill pathogenic organisms without harming normal human flora, thereby reducing the potential for resistance development. Advances in scientific research may further enhance these benefits by enabling the prediction and modification of depolymerase structures and the development of cocktails targeting different capsules [52–54].

Previous studies have shown that bacteriophage depolymerases targeting capsules can weaken bacterial capsules, rescue infected mice, and improve survival rates [55,56]. Our study demonstrates that bacteriophage depolymerases targeting capsules are ineffective against hvKp growth *in vitro*. However, Dep1979 exhibited potent therapeutic effects against hvKp infection in immunocompetent mice, but its *in vivo* efficacy was greatly reduced in severely immunosuppressed mice. Considering that capsules can help bacteria evade host immunity, we constructed immunosuppressed mice to compare treatment outcomes between immunosuppressed and immunocompetent mice, exploring the importance of host immunity to assess the *in vivo* antibacterial activity of depolymerases. Our results show that the 7-day survival rate of immunosuppressed mice infected with Dep1979 at an initial bacterial load of 10^6^ CFU was significantly lower than that of immunocompetent mice. However, when the initial bacterial load was 10^5^ CFU, Dep1979 could still rescue infected immunosuppressed mice, possibly because cyclophosphamide only inhibits most immune cells, while residual and regenerating immune cells remain functional [29,57].

Overall, our research highlights that bacteriophage depolymerases exhibit varying efficacy in treating different immune states of mice infected with hvKp. Furthermore, removing the capsule of hvKp is crucial for effective treatment.

## Conclusions

In this study, the therapeutic effects of phage depolymerase in immunocompetent and immunosuppressed mice were compared. In immunocompetent mice, Dep1979 effectively rescued mice infected with hvkp at lower treatment doses (0.1 mg/kg) while reducing systemic inflammatory response and tissue damage. In immunosuppressed mice, Dep1979 could only rescue mice with lower initial bacterial loads. These findings highlight the critical role of host immunity in the effectiveness of phage depolymerase and suggest that phage depolymerase holds potential as a treatment for hvkp infections.

## Supplementary Material

Supplementary Figure 1.tif

Author Checklist E10 only.pdf

## Data Availability

The authors declare that all data supporting the findings of this study are available in the paper. The data set linked with this submission can be found at https://www.scidb.cn/en/s/aUjMfa
