# Supplementary figures and images for: Synergy of bacteriophage depolymerase with host immunity rescues sepsis mice infected with hypervirulent Klebsiella pneumoniae of capsule type K2
Source: Virulence. 2024 Oct 21;15(1):2415945. doi: 10.1080/21505594.2024.2415945 (PMC11497950; doi:10.1080/21505594.2024.2415945)

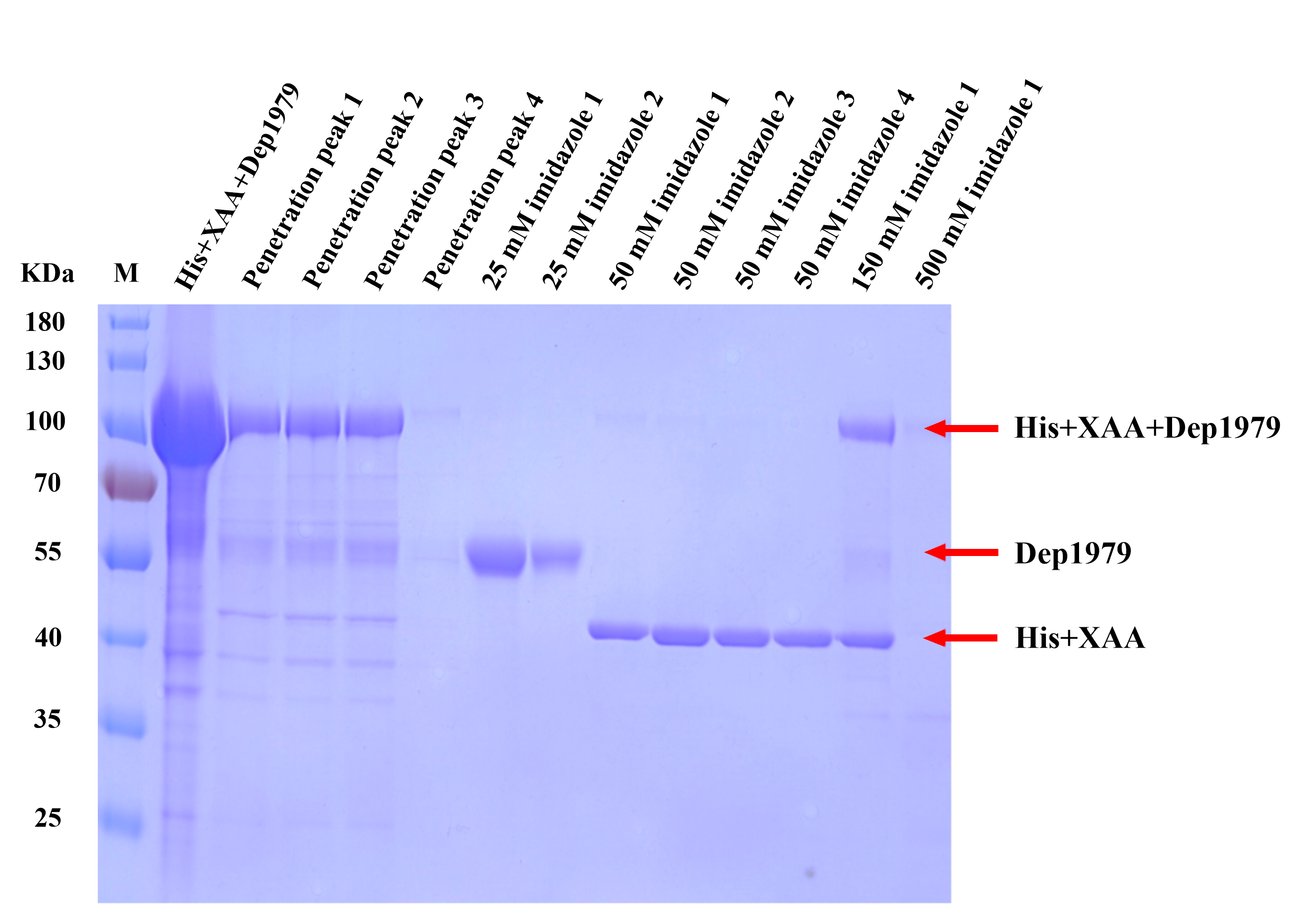

Supplement: Supplementary Figure 1.tif [file KVIR_A_2415945_SM2142.tif]
